# Prognostic Value of Primary Total Glossectomy in Tongue Cancer: A Systematic Review and Meta-Analysis of Survival Outcomes †

**DOI:** 10.3390/diagnostics15222847

**Published:** 2025-11-10

**Authors:** M. P. Sreeram, Prajwal Dange, Karthik N. Rao, Luiz P. Kowalski, Remco de Bree, Orlando Guntinas-Lichius, Alfio Ferlito

**Affiliations:** 1 Department of Head and Neck Oncology, Sri Shankara Cancer Hospital and Research Centre, Bangalore 560004, India; drsreeram111@gmail.com (M.P.S.); prajwal.dange@gmail.com (P.D.); 2 Head and Neck Surgery and Otorhinolaryngology Department, A. C. Camargo Cancer Center, Sao Paulo 01509-900, Brazil; lp_kowalski@uol.com.br; 3 Head and Neck Surgery, Faculty of Medicine, University of Sao Paulo, Sao Paulo 05508-020, Brazil; 4 Department of Head and Neck Surgical Oncology, University Medical Center Utrecht, 3508 GA Utrecht, The Netherlands; r.debree@umcutrecht.nl; 5 Department of Otorhinolaryngology, Jena University Hospital, 07740 Jena, Germany; orlando.guntinas@med.uni-jena.de; 6 Coordinator of the International Head and Neck Scientific Group, 35100 Padua, Italy; profalfioferlito@gmail.com

**Keywords:** advanced oral cavity cancer, total glossectomy, oncological outcomes, survival outcomes, overall survival, morbidity, incidence meta-analysis, compartment tongue surgery

## Abstract

**Background/Objectives:** Total glossectomy (TG) is among the most radical operations in head and neck oncology. While it can achieve local control in advanced oral tongue squamous cell carcinoma, survival and functional outcomes are inconsistently reported, and pooled estimates remain limited. This study aimed to systematically evaluate survival, functional recovery, and prognostic factors following primary TG. **Methods:** We conducted a proportional meta-analysis of studies reporting outcomes after primary TG for oral tongue squamous cell carcinoma. Studies combining TG with laryngectomy, salvage settings, or second primary tumors were excluded. Two reviewers independently screened, extracted data, and assessed quality with the Newcastle–Ottawa Scale. Pooled 1-, 3-, and 5-year overall survival (OS) with 95% confidence intervals (CIs) was calculated using a random-effects model. Heterogeneity was quantified (Q, τ^2^, I^2^), and robustness was assessed with sensitivity analyses. Disease-free survival (DFS) and functional outcomes (swallowing, airway, speech) were narratively summarized due to inconsistent reporting. **Results**: Ten studies (1992–2022) comprising 261 patients met the criteria. Pooled OS was 81% (95% CI, 71–90) at 1 year, 55% (95% CI, 41–68) at 3 years, and 47% (95% CI, 27–67) at 5 years, with rising heterogeneity (I^2^ up to 89%). The post-2000 series showed improved 5-year OS (63%). Adverse prognostic factors included advanced T stage, nodal disease (N+), and positive margins. Functional recovery varied: 15–30% remained gastrostomy-dependent and 20–25% aspirated, while reconstruction and structured rehabilitation improved outcomes. **Conclusions**: Survival after TG declines beyond the first year, with under half surviving at 5 years, though modern outcomes appear better. Significant functional morbidity underscores the need for multidisciplinary care. Future biomarker-driven studies should refine patient selection and prognostic assessment.

## 1. Introduction

Oral tongue squamous cell carcinoma represents one of the most aggressive malignancies of the head and neck, accounting for approximately 1% of all cancers in the United States, with a disproportionately higher incidence in developing countries due to risk factors such as tobacco and betel quid chewing [1]. Surgery remains the primary curative modality, ranging from partial to total glossectomy, often combined with adjuvant radiotherapy or chemoradiation. The tongue’s compartmental anatomy promotes an aggressive pattern of tumor progression along muscle fibers and neurovascular channels, contributing to its poor prognostic biology. Total glossectomy, however, is among the most radical procedures in head and neck oncology, representing the most extensive resection along a spectrum that includes partial, hemi-, and subtotal glossectomy, each with distinct functional and prognostic implications. This procedure results in the complete loss of the tongue, an organ critical for speech articulation, swallowing propulsion, and airway protection. While it may achieve local control in patients with extensive or recurrent disease, it carries profound implications for swallowing, airway, and communication, and is frequently associated with long-term morbidity [2]. Recent systematic analyses have quantified these functional burdens: pooled data indicate that approximately 23% of patients remain feeding-tube-dependent, 7% require permanent tracheostomy, yet 91% can achieve intelligible speech, though overall complication rates approach 33% [3].

Despite being performed for decades, the true oncologic benefit of TG remains debated. Reported survival outcomes vary widely across single-institution series, influenced by disease stage, indication for surgery, and prior treatments, particularly for salvage after failed organ-preservation protocols [4,5]. Functional outcomes are similarly heterogeneous, with persistent gastrostomy or tracheostomy dependence reported in a significant subset of survivors. Advances in reconstructive techniques and perioperative care may have improved contemporary results, yet survival rates remain below 50% at five years in many series [6].

Given the rarity and radicality of this procedure and the absence of prospective trials, robust pooled estimates of survival and functional outcomes are lacking. A systematic review and meta-analysis are, therefore, essential to better inform surgical decision-making, patient counseling, and long-term care planning. This meta-analysis systematically evaluates prognostic factors and diagnostic criteria associated with survival and functional outcomes following primary TG, thereby providing a benchmark for clinical prognosis and against which future therapeutic innovations, including reconstructive advances and systemic therapies such as immunotherapy, can be assessed.

## 2. Materials and Methods

### 2.1. Study Design and Reporting Guidelines

This meta-analysis was conducted and reported in accordance with the Preferred Reporting Items for Systematic Reviews and Meta-Analyses (PRISMA) statement guideline [7]. The methodological quality of this review was evaluated using the Assessment of Multiple Systematic Reviews (AMSTAR) checklist [8] to ensure adherence to established standards for systematic review conduct and reporting.

### 2.2. Literature Search Strategy

A comprehensive literature search was performed across multiple electronic databases, including PubMed, Scopus and Google Scholar, to identify all relevant studies reporting survival outcomes following TG procedures. The search strategy covered publications from inception to 26 June 2025, without language restrictions initially, though only English-language studies were ultimately included in the final analysis.

The search strategy employed a combination of medical subject headings and free-text terms related to the population of interest and outcomes. Population terms included “glossectomy,” “total glossectomy,” “complete glossectomy,” “tongue resection,” “oral cancer surgery,” and “tongue cancer.” Outcome-related terms encompassed “survival,” “mortality,” “prognosis,” “follow-up,” “overall survival,” and “disease-free survival.” These terms were combined using appropriate Boolean operators (AND, OR) to maximize sensitivity while maintaining reasonable specificity (Table S1 in Supplementary Material). Reference lists of identified studies and relevant systematic reviews were manually searched to identify additional potentially eligible studies that may have been missed in the electronic search.

### 2.3. Study Selection and Eligibility Criteria

Two independent reviewers (PD and SMP) conducted the study selection process in two stages. Initially, titles and abstracts of all retrieved citations were screened against predetermined eligibility criteria. Subsequently, full-text articles of potentially relevant studies were obtained and assessed for final inclusion. Any disagreements between reviewers were resolved through discussion, and when necessary, a third senior reviewer (KNR) was consulted to reach consensus.

Studies were considered eligible for inclusion if they reported survival outcomes in adult patients who underwent TG procedures for primary squamous cell carcinoma. Studies are needed to provide sufficient data to calculate survival rates at specified time intervals, with adequate follow-up duration documented. Only peer-reviewed publications were considered eligible, and studies needed to clearly define their patient population and surgical procedures.

Studies were excluded if they focused solely on partial glossectomy procedures, if outcomes for TG patients could not be separated from mixed surgical cohorts, if the procedure was combined with glosso-laryngectomy, or if TG was performed as salvage surgery or for second primary tumors. Conference abstracts, case reports, and studies lacking adequate survival data or follow-up information were excluded from the analysis. Studies that did not provide sufficient methodological detail to assess quality or extract relevant data were also excluded.

### 2.4. Data Extraction Process

Two independent reviewers (PD and SMP) extracted data from each included study, with discrepancies resolved through discussion or consultation with a third reviewer (KNR) when necessary. The extraction process captured comprehensive study characteristics, including study design, geographic location, study period, and institutional setting.

Patient-level data extracted included sample size, demographic characteristics such as age and gender distribution, tumor characteristics including primary site location, and TNM staging information where reported. Surgical details encompassed the specific surgical approach used, the extent of resection, the reconstruction methods employed, and any concurrent procedures performed.

For studies that included both total and subtotal glossectomy patients, data extraction focused specifically on patients who underwent TG procedures, with outcomes separated from subtotal glossectomy cases where possible. Only patients undergoing TG as primary treatment were considered for analysis. Patients who underwent TG for second primary tumors or salvage surgery following previous treatment failures were excluded from data extraction.

Survival data were extracted primarily from reported survival rates (OS and DFS) at one, three, and five-year intervals following surgery. When survival rates were not directly reported, Kaplan–Meier survival curves were analyzed using digital plot digitization software to extract survival estimates at the specified time points. The number of events (deaths) and patients at risk were recorded for each study to calculate pooled survival estimates. While disease-free survival (DFS) data were also collected and reported for descriptive purposes, the quantitative meta-analysis was conducted exclusively on OS.

### 2.5. Quality Assessment

#### 2.5.1. Level of Evidence

The level of evidence in eligible studies was independently assessed by two reviewers using the Oxford Centre for Evidence-Based Medicine (OCEBM) levels of evidence framework, with disagreements resolved through discussion.

#### 2.5.2. Methodological Quality

The methodological rigor of the selected studies was systematically evaluated using established quality assessment tools appropriate for observational studies. Two independent reviewers evaluated each study using standardized criteria, with disagreements resolved through discussion. The assessment focused on key methodological domains, including patient selection procedures, comparability of study groups where applicable, adequacy of outcome assessment methods, and completeness of follow-up data.

The methodological quality of included studies was systematically assessed using the Newcastle–Ottawa Scale (NOS) for observational studies [9]. The NOS evaluates studies across three domains: selection of study participants (4 items), comparability of cohorts (1 item), and assessment of outcomes (3 items), with a maximum score of 9 stars. Two independent reviewers evaluated each study using the standardized NOS criteria, with disagreements resolved through discussion or consultation with a third reviewer when necessary.

The assessment focused on key methodological domains, including patient selection procedures and representativeness of the study population, comparability of study groups where applicable, adequacy of outcome assessment methods, including ascertainment of survival outcomes, and completeness of follow-up data. Studies scoring 7–9 stars were considered high quality, 4–6 stars moderate quality, and 0–3 stars low quality for the purposes of sensitivity analysis.

Specific attention was paid to the clarity of inclusion and exclusion criteria, representativeness of the study population relative to patients undergoing total glossectomy, adequacy of sample size for survival analysis, and appropriateness of statistical methods employed for survival analysis, including handling of censored data. The assessment also considered potential sources of bias, including selection bias in patient recruitment, information bias in outcome ascertainment, loss to follow-up rates, and confounding variables that may have influenced study results.

Individual study quality scores were tabulated, and studies with a high risk of bias (NOS score < 4) were considered for exclusion in sensitivity analyses to assess the robustness of pooled survival estimates. The overall quality of evidence across studies was considered in the interpretation of meta-analysis results as well as an assessment of the evidence.

### 2.6. Statistical Analysis Methods

#### 2.6.1. Primary Analysis

The primary analysis involved pooling overall survival proportions across included studies using random-effects meta-analysis methodology [10]. Pooled survival proportions with corresponding 95% confidence intervals were calculated for one, three, and five-year survival endpoints.

#### 2.6.2. Heterogeneity Assessment

Statistical heterogeneity was evaluated using the DerSimonian–Laird estimator to determine between-study variance (tau^2^), quantifying variations in true effect sizes among studies. The Cochran Q-test was applied to examine significant heterogeneity based on the comparison of individual study effect sizes with the overall pooled estimate [11]. The Higgins I^2^ statistic was calculated to express the proportion of total variability attributable to heterogeneity as a percentage, with 0% indicating no observed heterogeneity and higher values representing increased heterogeneity. Values exceeding 50% were considered indicative of substantial heterogeneity. Prediction intervals were calculated when heterogeneity was detected (tau^2^ > 0) to accommodate uncertainty arising from between-study variation [12].

#### 2.6.3. Effect Size Visualization

Forest plots were constructed to display individual study estimates alongside pooled effects with confidence intervals. L’Abbé plots were generated to visualize the relationship between survival rates across different time points and assess patterns of heterogeneity [13].

#### 2.6.4. Sensitivity and Subgroup Analyses

Leave-one-out sensitivity analysis [14] was performed by systematically excluding each study and recalculating pooled estimates to identify studies with disproportionate influence on results. Studentized residuals were calculated to identify potential outliers, with studies exceeding predetermined thresholds visualized using radial plots. Subgroup analyses examined the impact of study characteristics, including sample size and publication period, on survival estimates.

#### 2.6.5. Influential Study Assessment

Cook’s distances were computed to quantify each study’s impact on the overall meta-analysis model. Studies with Cook’s distances exceeding the calculated threshold were considered potentially influential on pooled estimates [15].

#### 2.6.6. Publication Bias Evaluation

Assessment of publication bias was limited by the small number of included studies (*n* = 10). Visual inspection of funnel plots was performed, though interpretation was cautious given that statistical tests for publication bias have limited power with fewer than 15 studies. The potential impact of unpublished studies on pooled estimates was considered qualitatively in the discussion of study limitations [16].

#### 2.6.7. Statistical Software

All analyses were conducted using R statistical software version 4.5.1 with specialized meta-analysis packages. Statistical significance was set at *p* < 0.05 for all analyses except heterogeneity assessment, where *p* < 0.10 was used as the significance threshold [17].

## 3. Results

### 3.1. Literature Retrieval and Data Extraction

**Figure 1:**
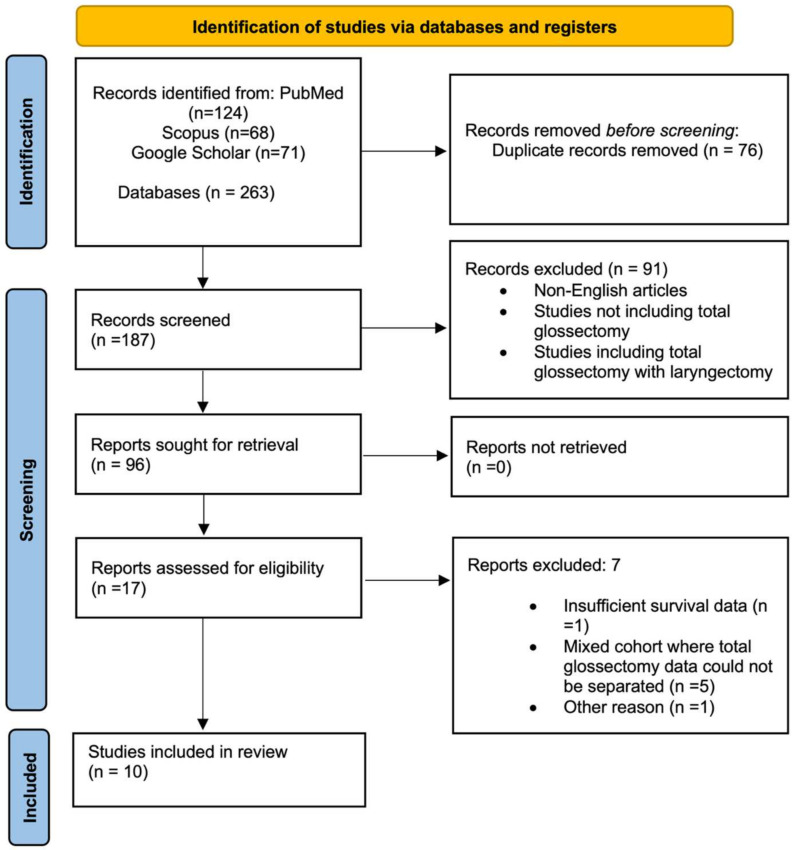
PRISMA Flow diagram.

The initial literature search identified 263 articles across three databases (Figure 1). PubMed yielded 124 articles, Scopus contributed 68 articles, and Google Scholar provided 71 articles. After removing 76 duplicates, 187 articles remained for title and abstract screening. Following initial screening, 91 articles were excluded for various reasons, including non-English language, studies not focusing on TG, or studies combining TG with laryngectomy procedures. This resulted in 96 articles proceeding to full-text assessment.

During full-text evaluation, 17 articles were identified that contained survival data for TG patients. After applying strict inclusion criteria requiring studies to report survival outcomes specifically for primary TG in squamous cell carcinoma of the tongue, 10 studies [18,19,20,21,22,23,24,25,26,27] were ultimately included in the final meta-analysis. Seven studies were excluded during full-text review due to insufficient survival data, inability to separate TG outcomes from mixed cohorts, or failure to meet methodological quality standards.

### 3.2. Study Characteristics and Quality Assessment

The meta-analysis comprised 10 studies published between 1992 and 2022, encompassing survival data from different geographic regions, including the United States (4 studies) [18,20,22,26], Europe (3 studies) [23,24,25], Asia (1 study) [27], Brazil (1 study) [19], and Australia (1 study) [21]. The studies included a total of 261 patients who underwent TG for primary squamous cell carcinoma of the tongue. Sample sizes ranged from 7 to 75 patients, with a median study size of 24 patients. Individual study details are provided in Table 1. The survival data were extrapolated from Kaplan–Meier curves and reported for each year, whereas functional outcomes were documented only for upfront primary total glossectomy. Moreover, in most studies, salvage procedures were combined with upfront surgeries, limiting the ability to distinctly assess their individual impact.

Quality assessment using the Newcastle–Ottawa Scale revealed generally high methodological quality across included studies. Eight studies (80%) achieved high quality ratings (≥7 stars), while two studies (20%) received moderate quality ratings (4–6 stars). No studies were classified as low quality. The assessment identified adequate patient selection procedures and outcome ascertainment in most studies, though some limitations were noted in comparability domains due to the observational nature of the included studies.

### 3.3. Publication Bias Assessment

**Figure 2:**
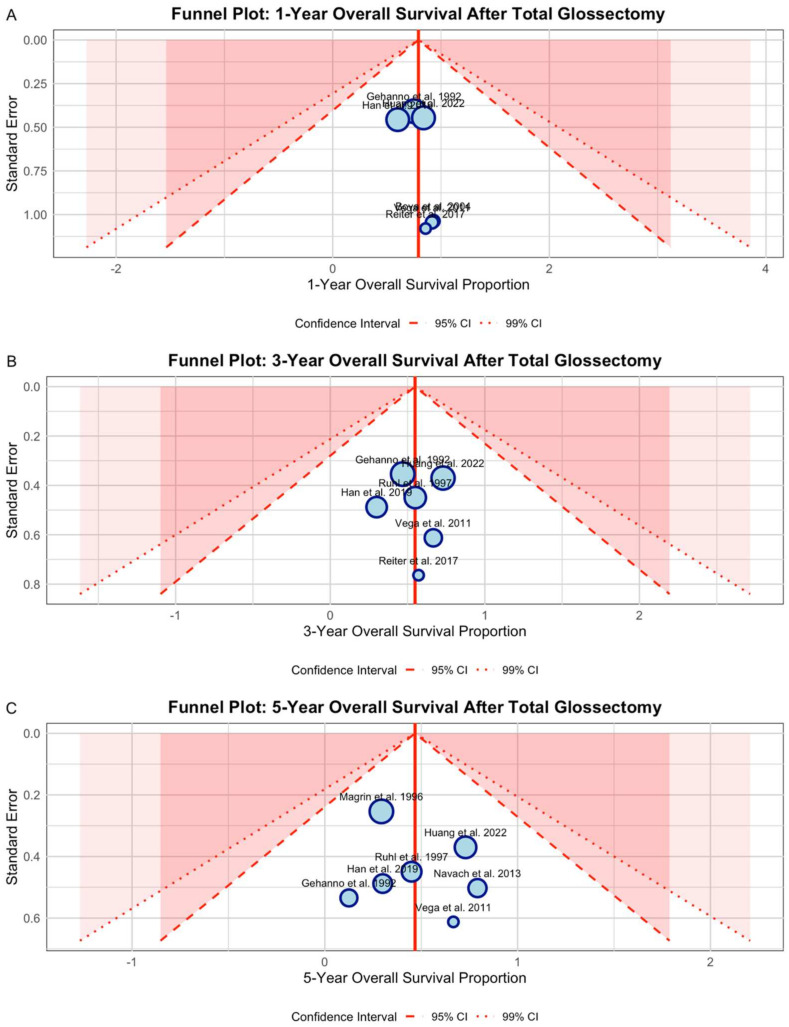
Funnel plots for publication bias assessment. (**A**) One-year survival from 6 studies (122 patients): symmetric distribution around the pooled estimate. (**B**) Three-year survival from 6 studies (128 patients): studies within confidence boundaries despite moderate heterogeneity. (**C**) Five-year survival from 7 studies [18,19,20,23,24,26,27] (220 patients): no evidence of publication bias despite substantial heterogeneity.

Visual inspection of funnel plots for 1-year, 3-year, and 5-year survival showed studies reasonably distributed around pooled estimates without systematic asymmetry (Figure 2). The 5-year survival funnel plot demonstrated the greatest scatter, reflecting the substantial heterogeneity observed in quantitative analysis (I^2^ = 85.5%). Studies fell within and outside the 95% and 99% confidence regions without preferential clustering in areas of statistical significance. While some studies appeared as outliers (particularly Han et al. [26] for intermediate-term survival and Gehanno et al. [18] for long-term survival), the overall patterns were consistent with genuine between-study variation rather than systematic publication bias.

#### 3.3.1. One-Year Overall Survival

**Figure 3:**
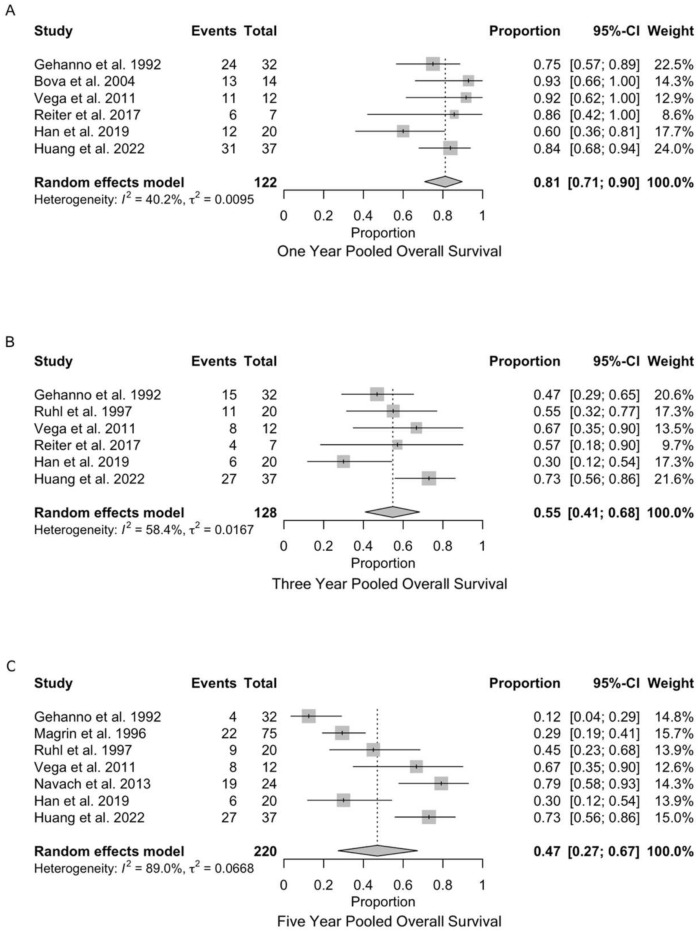
Forest plots of overall survival after total glossectomy. (**A**) One-year: pooled estimate 81% (95% CI: 71–90%, I^2^ = 40.2%) [18,21,23,25,26,27]. (**B**) Three-year: pooled estimate 55% (95% CI: 41–68%, I^2^ = 58.4%) [18,20,23,25,26,27]. (**C**) Five-year: pooled estimate 47% (95% CI: 27–67%, I^2^ = 89.0%) [18,19,20,23,24,26,27].

One-year overall survival data were available from 6 studies encompassing 122 patients (Figure 3A). The pooled one-year overall survival rate was 81% (95% CI: 71–90%), indicating that approximately 4 out of 5 patients survived the first year following total glossectomy. Statistical heterogeneity was moderate with I^2^ = 40.2% and τ^2^ = 0.0095, suggesting some variability between studies but not substantial enough to undermine the pooled estimate. Individual study survival rates ranged from 60% [26] to 93% [21], a 33-percentage point difference reflecting variability in patient populations, staging, treatment approaches, and institutional expertise across studies.

#### 3.3.2. Three-Year Overall Survival

Three-year overall survival data were extracted from 6 studies, including 128 patients (Figure 3B). The pooled three-year survival rate was 55% (95% CI: 41–68%), representing a substantial decline from one-year survival and indicating that fewer than 3 out of 5 patients survive to three years. Moderate heterogeneity was observed with I^2^ = 58.4% and τ^2^ = 0.0167, approaching the threshold for substantial heterogeneity and suggesting meaningful differences between study populations or methodologies. Individual study estimates ranged from 30% [26] to 73% [27], a remarkable 43 percentage point spread indicating substantial variation in intermediate-term survival outcomes.

#### 3.3.3. Five-Year Overall Survival

Five-year overall survival data were available from 7 studies comprising 220 patients (Figure 3C). The pooled five-year survival rate was 47% (95% CI: 27–67%), demonstrating that fewer than half of patients survive five years following total glossectomy. Substantial heterogeneity was present with I^2^ = 89.0% and τ^2^ = 0.0668, indicating significant between-study variation that limits confidence in the pooled estimate and suggests important differences in study characteristics or patient populations. Individual study survival rates varied considerably from 12% [18] to 79% [24], representing a striking 67 percentage point range that reflects significant between-study variation in long-term outcomes.

### 3.4. Sensitivity Analysis

**Figure 4:**
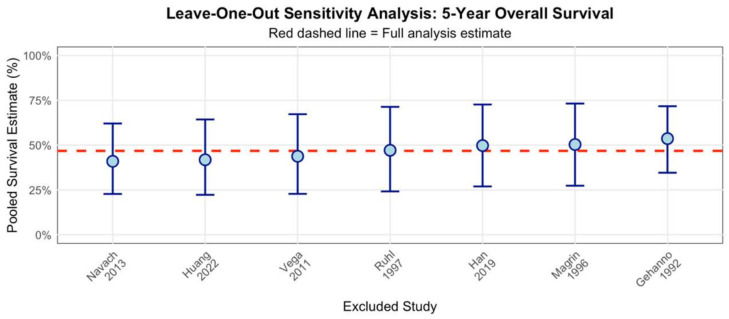
Leave-one-out sensitivity analysis for five-year overall survival [18,19,20,23,24,26,27].

Leave-one-out sensitivity analysis was performed for the five-year overall survival endpoint to assess the robustness of the pooled estimate and identify potentially influential studies (Figure 4). Systematic exclusion of individual studies resulted in pooled estimates ranging from approximately 40% to 55%, with the lowest estimate observed after excluding Navach et al. [24] and the highest after excluding Gehanno et al. [18]. The maximum absolute difference from the full analysis estimate was approximately 8 percentage points, with all confidence intervals overlapping substantially, confirming that no single study exerted disproportionate influence on the overall pooled estimate of 47%. Despite the substantial heterogeneity (I^2^ = 89.0%) observed in the five-year survival data, the sensitivity analysis demonstrated acceptable stability of the meta-analysis results, suggesting that the heterogeneity reflects genuine clinical diversity rather than methodological bias or undue influence from outlying studies.

### 3.5. Post-2000 Sensitivity Analysis

**Figure 5:**
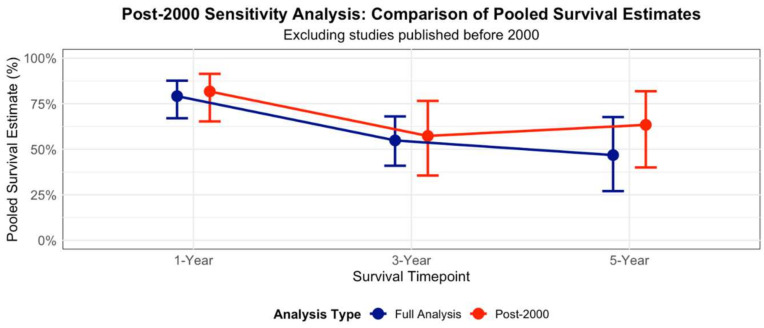
Post-2000 Sensitivity analysis: Comparison of pooled estimates.

A post-2000 sensitivity analysis was performed to assess whether older studies published before 2000 influenced the pooled survival estimates (Figure 5). For one-year survival, excluding [18] resulted in a pooled estimate of 84% (95% CI: 68–93%) compared to 81% (95% CI: 71–90%) in the full analysis, representing a minimal increase of 3 percentage points. For three-year survival, excluding [18,20] yielded an estimate of 59% (95% CI: 35–79%) compared to 55% (95% CI: 41–68%), showing a 4-percentage point difference. The most substantial change was observed in five-year survival, where exclusion of three pre-2000 studies [18,19,20] resulted in a pooled estimate of 64% (95% CI: 39–83%) compared to 47% (95% CI: 27–67%), representing a notable 17 percentage point increase. Despite these changes, confidence intervals overlapped substantially between analyses, and the post-2000 analysis maintained considerable heterogeneity (I^2^ values remained elevated). These findings suggest that while more recent studies trend toward improved survival outcomes, particularly at five years, the overall meta-analysis conclusions remain generally robust across different publication eras.

### 3.6. Disease-Free Survival

Disease-free survival data were available from only 4 of the 10 included studies [21,22,24,27], representing a significant limitation in outcome reporting. One-year DFS data were extractable from 3 studies [21,22,27] encompassing 71 patients, with rates ranging from 40% [22] to 92.9% [21], and an intermediate rate of 64% reported by Huang et al. [27]. Two-year DFS was available from only Sinclair et al. [22] showing a decline to 35%, while 3-year DFS was reported solely by Huang et al. [27] at 57%. Five-year DFS outcomes were available from 3 studies [21,24,27], including 75 patients, with rates ranging from 28.6% [21] to 61% [24].

The limited reporting of DFS outcomes across most studies (60% did not report DFS) represents a critical gap in the literature, as disease-free survival is an essential endpoint for evaluating oncologic efficacy of TG, particularly given the significant morbidity associated with salvage treatments following recurrence.

### 3.7. Heterogeneity Assessment and L’Abbé Plot Analysis

**Figure 6:**
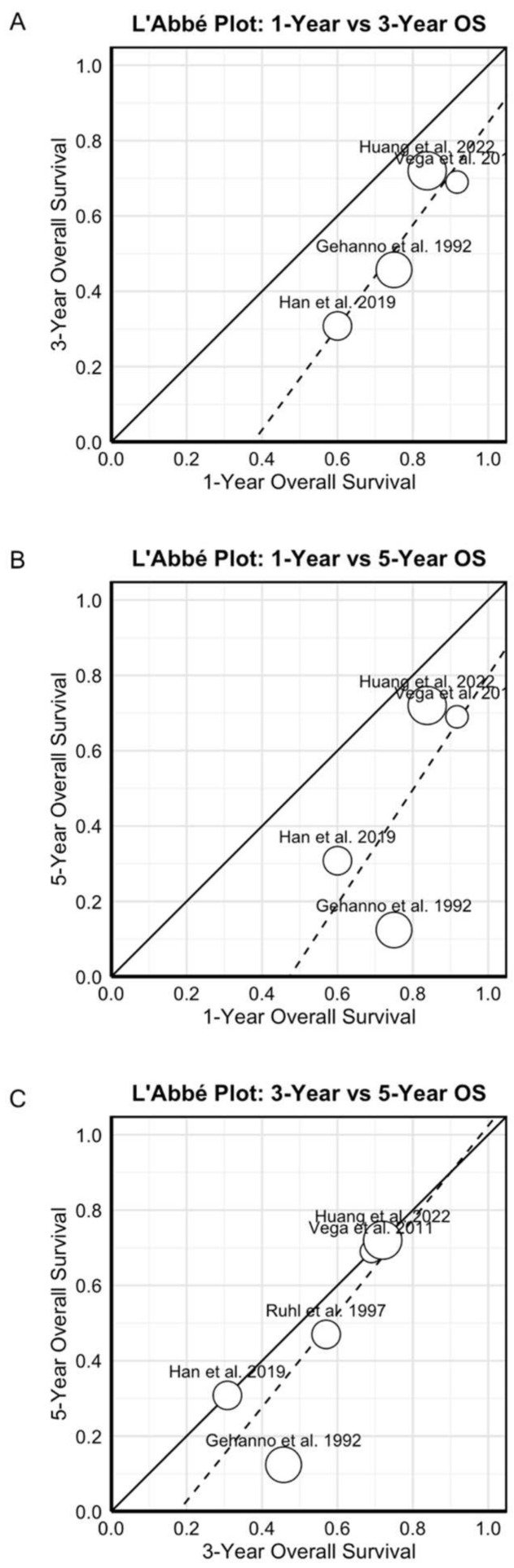
L’Abbé plots showing survival trends over time. (**A**) One-year versus three-year survival [18,23,26,27]. (**B**) One-year versus five-year survival [18,23,26,27]. (**C**) Three-year versus five-year survival [18,20,23,26,27]. All studies fall below the equality line, confirming progressive mortality, with stabilization after three years (Recent publications show improvement in 5-year OS trend). The solid diagonal line represents the line of equality (y = x), where survival rates at both time points would be identical. The dotted line indicates the fitted regression line showing the observed relationship between survival rates at the two time points. Points below the solid line indicate a decline in survival over time, with the size of the circle proportional to the study’s sample size (or weight) in the analysis.

L’Abbé plots demonstrated clear patterns of survival decline over time (Figure 6). The 1-year versus 5-year survival plot showed all studies positioned below the line of equality, confirming expected mortality progression (Figure 6B). Similarly, the 1-year versus 3-year and 3-year versus 5-year comparisons revealed consistent patterns of survival deterioration, though with varying trajectories across studies (Figure 6A,C). These visualizations highlighted the substantial between-study heterogeneity observed in the quantitative analyses, particularly for longer-term survival endpoints. The progressive increase in heterogeneity from one-year (I^2^ = 40.2%) to five-year survival (I^2^ = 89.0%) suggests that factors influencing long-term outcomes may vary more substantially across studies than those affecting short-term survival.

### 3.8. Functional Outcomes

Functional outcome data were available from 5 of the 10 included studies [21,22,23,24,25], though most studies did not separate outcomes for primary versus salvage surgery cases. Swallowing outcomes demonstrated considerable variation across studies, ranging from complete feeding tube dependency in Sinclair et al.’s TG cohort to 90% independent oral feeding reported by Bova et al. Among studies reporting data for primary cases or mixed cohorts, oral feeding independence rates ranged from 70% [24] to 85% [23], with Reiter et al. [25] reporting that 78.6% of patients resumed oral feeding without gastrostomy dependency. Tracheostomy decannulation rates were similarly variable, with Sinclair et al. [22] reporting 50% permanent tracheostomy dependency in their TG cohort, while other studies achieved decannulation rates of 85.7% to 90% in patients with laryngeal preservation. As glosso-laryngectomy cases were excluded from this review, these figures reflect only TG with laryngeal preservation, including oral tongue lesions with extension into the base of the tongue. Speech outcomes were generally more favorable, with intelligible speech achieved in 87% to 100% of patients in larynx-preserving series, although only 30% of patients in Sinclair et al.’s TG cohort achieved functional speech. The substantial variation in functional outcomes likely reflects differences in surgical approach, particularly regarding laryngeal preservation, patient selection criteria, and the proportion of primary versus salvage cases within study cohorts. Studies that included laryngeal preservation [21,23,25] consistently reported superior speech and swallowing outcomes compared to those performing TG with laryngectomy. The limited separation of primary and salvage surgery outcomes in most studies represents a significant limitation, as salvage cases typically present with worse baseline function and more challenging reconstruction requirements. The heterogeneity in outcome definitions and assessment methods across studies precluded meaningful meta-analysis of functional endpoints, highlighting the need for standardized functional outcome reporting in future TG research.

## 4. Discussion

### 4.1. Survival Outcomes

**Figure 7:**
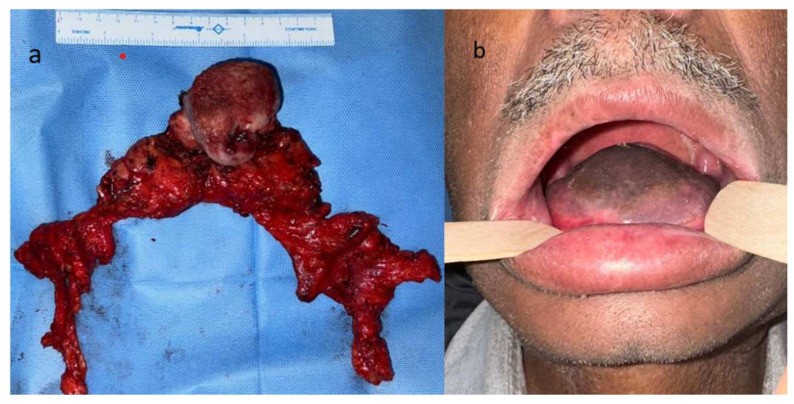
(**a**) Resected specimen following TG with bilateral lymph node dissection from levels I to V. (**b**) Reconstruction with anterolateral thigh (ALT) flap demonstrating the required optimal bulk for achieving improved functional outcomes.

Advanced tumors of the oral tongue profoundly affect survival, function, and quality of life [28]. Management typically involves either primary surgical resection with adjuvant radiotherapy or chemoradiation, or definitive radiation or chemoradiation therapy. Total glossectomy, though rarely performed, may be considered in select cases, particularly when tumors are extensive and refractory to other treatment modalities [29,30,31,32,33,34]. A specimen of a TG with en bloc nodal clearance is shown in Figure 7.

The procedure is inherently lengthy, aggressive, and associated with substantial morbidity. Reconstruction, often attempted with a variety of local or free flap designs, has not consistently demonstrated significant improvements in long-term functional outcomes. Consequently, the decision to proceed with TG requires careful multidisciplinary deliberation, including the tumor board, reconstructive and rehabilitation teams, and, critically, the fully informed consent of the patient and family. The choice must originate from the patient’s own motivation, values and preferences, given that the surgery frequently results in long-term or lifelong dependence on supportive measures and diminished quality of life.

Despite advances in surgical technique and perioperative care, overall survival following TG remains limited. Surgical resection is frequently the preferred approach for oral cavity cancer, especially in advanced stages or as a salvage procedure after failed organ preservation protocols such as neoadjuvant chemotherapy/chemoradiotherapy. Retrospective clinical series evaluating TG reveal a broad range of survival outcomes, dictated largely by patient selection, disease stage, and indication for surgery.

Survival outcomes following TG vary widely across reported series but consistently illustrate the potential for meaningful disease control in carefully selected patients. Bova et al. observed excellent short-term outcomes in a small Australian cohort, with 1-year OS of 92.9%, though only 28.6% remained disease-free at 5 years [21]. Gehanno et al., in a larger series of 32 patients, reported 1-year OS of 74.1%, but long-term survival was poor, with 3-year and 5-year OS declining to 45.7% and 12.4%, respectively [18]. Han et al. likewise reported attrition over time, with OS decreasing from 62% at 1 year to approximately 30% at both 3 and 5 years. In contrast, Huang et al. documented strikingly more favorable outcomes in their Taiwanese cohort, with 3- and 5-year OS both at 72% and 5-year DFS of 57%, suggesting the possibility of durable disease control in certain populations [27]. Similarly, Magrin et al. noted 2-year OS of 39% and 5-year OS of 29% among 75 patients, underscoring the high risk of recurrence in large series [19].

Other reports highlight both the heterogeneity of patient selection and the impact of surgical extent on long-term prognosis. Navach et al. achieved some of the best reported outcomes, with 5-year DFS and OS of 61% and 79%, respectively, in 24 patients [24]. Reiter et al., though limited by a cohort of only 7, showed a 3-year OS of 57.1% but a sharp decline to 14.3% by 4 years [25]. Ruhl et al. found 3- and 5-year OS rates of 57% and 47% in their survival cohort, with better results in patients treated without laryngectomy [20]. Sinclair et al. demonstrated early relapses, with 2-year DFS of 35% and 3-year DFS of 40%, while Vega et al. reported consistently favorable survival of 69% at both 3 and 5 years [22,23]. Collectively, these data reveal 5-year OS estimates ranging from as low as 12% to nearly 80%, reflecting both the aggressive nature of advanced oral squamous cell carcinoma and the potential for long-term survival in a subset of patients. A recent large retrospective cohort analyzing radical surgery with adjuvant therapy in advanced oral tongue cancer reported a 3-year overall survival of 49.2 and median survival of 15.6 months, with nodal metastasis significantly reducing median survival from 22.0 months in N0 disease to 12.9 months in node-positive cases, underscoring the critical prognostic impact of nodal status [35].

This study’s pooled incidence meta-analysis reveals that OS rates following TG declined consistently with extended follow-up duration. Initially, the pooled 1-year OS rate was 81%, based on data compiled from 6 studies involving 122 patients. This survival estimate decreased significantly to 55% at 3 years, based on 6 studies covering 128 patients. At a longer follow-up, the pooled 5-year OS rate further declined to 47%, incorporating data from 7 studies involving 220 patients.

A crucial finding, however, is the improvement in long-term survival when focusing solely on more recent literature: when analyzing 4 studies published after the year 2000, the pooled 5-year OS rate improved significantly to 63%. This demonstrable improvement in survival outcomes since the turn of the century suggests that progress in the management of advanced tongue cancer may be linked to significant advancements in both adjuvant therapies and specialized surgical technologies [36].

A key limitation is the potential for publication bias, which could lead to an overestimation of the true survival benefit. This meta-analysis relies exclusively on published case series, which may reflect institutional expertise and are more likely to report favorable outcomes. If smaller studies with poorer results remain unpublished, our pooled estimates, particularly the 5-year overall survival of 47%, may be optimistically biased. While funnel plot analysis did not reveal significant asymmetry, the limited number of included studies (*n* = 10) means we cannot definitively rule out this bias. Therefore, the results should be interpreted as representing the best available evidence from published literature rather than the absolute outcome for all patients undergoing this procedure.

### 4.2. Prognostic Significance of Treatment Indication and Disease Stage

A critical determinant of oncological outcome is whether TG is performed as primary treatment or as salvage therapy following failure of previous modalities, particularly chemoradiotherapy. Navach et al. analyzed 37 patients who underwent TG with laryngeal preservation and found a dramatic difference: for previously untreated patients (*n* = 24), the 5-year OS was 79% and 5-year DFS was 61%; however, for patients undergoing salvage glossectomy (*n* = 13), the 5-year OS was 21% and 5-year DFS was 23% [24]. Similarly, high survival was reported by Huang et al., using a novel technique (sternocleidomastoid flap reconstruction), who achieved an overall five-year survival of 72% [27]. Vega et al. also observed that the 5-year actuarial survival rate for primary neoplasia was 69%, but it was drastically reduced to 27% for salvage surgical procedures. The high percentage of salvage cases, especially in series dealing with advanced (Stage IV) disease, often correlates with poorer overall results [23]. For instance, Han et al., reviewing 48 cases where 60.4% were salvage procedures, reported lower 1- and 5-year survival rates of 42% and 26%, respectively [26]. Conversely, a high percentage of salvage surgery (83%) in Mazarro et al.’s series led to a 3-year OS of only 25% [37].

Furthermore, the anatomical extent of the disease (T stage) significantly impacts survival outcomes. Gourin et al.’s study on base of tongue SCC demonstrated that survival sharply decreased with increasing T stage: the 5-year disease-specific survival rate was 88% for T1 lesions but dropped to 30% for T4 lesions [38]. Magrin et al. reported 5-year actuarial survival rates of 49% for T3 tumors and 20% for T4 tumors [19]. The extent of lymph node involvement and margin status are equally critical. The presence of positive or close margins, a frequent finding in TG patients (e.g., 60% in Bova et al. and 60% in Sinclair et al.), is consistently associated with a poor prognosis [21,22].

### 4.3. Functional Outcomes and Quality of Life Implications

TG survivors demonstrate a wide spectrum of swallowing and airway outcomes, but two consistent themes emerge: most patients eventually regain at least partial oral intake, while a substantial minority experience persistent aspiration risk or require ongoing enteral support. A comprehensive systematic review and meta-analysis pooling 642 patients reported feeding-tube dependence in 22.9%, tracheostomy dependence in 7.3%, and speech intelligibility in 91.1% at median 12-month follow-up, with an overall complication rate of 33.1%, including major fistula (3.0%) and aspiration pneumonia (2.8%) [3]. Large series and cross-sectional cohorts report that the majority of patients achieve feeding-tube independence, but reported rates vary by patient selection and setting. Bhattacharya et al. found roughly 85% feeding-tube independence (only three patients remained exclusively tube-dependent) and 68% of patients achieved mainly oral intake by FOIS criteria in their mixed glossectomy cohort [39]. Conversely, salvage cohorts show far higher long-term gastrostomy dependence. The GETTEC French multicenter study reported gastrostomy dependence at the end of follow-up of ~89% in the salvage TG group [40]. The largest reported case series of total glossectomy with total laryngectomy (TGTL) from a high-volume tertiary institution corroborated these findings, demonstrating that although most patients eventually achieved some restricted oral intake, the majority remained gastrostomy-dependent for primary nutrition, with partial oral feeding capacity often not regained until approximately 12 months postoperatively. These differences underscore that primary versus salvage setting, prior radiotherapy, and patient selection strongly influence feeding outcomes [41].

Airway management after TG is generally temporary for most patients but not uniformly brief. Many series report routine temporary tracheostomy with a median decannulation in the early postoperative weeks: in the long-term survivor cohort reported by Thaduri et al., median time to tracheostomy removal was 14 days (IQR 8; maximum 45 days) and no patients remained chronically tracheostomy-dependent at the time of assessment [42]. However, other series, particularly those including laryngeal involvement or extensive salvage resections, report higher and more prolonged tracheostomy rates (some historical series report dependence in up to ~50% of patients, and the GETTEC study found ~48% of TG patients had a tracheotomy at follow-up) [2,6,43,44,45,46,47,48]. In short, temporary tracheostomy is common and usually well tolerated, but long-term dependence is more likely in salvage or more extensive resections.

Aspiration and pharyngeal residue are frequent and clinically important sequelae. Objective endoscopic/fluoroscopic testing in these cohorts shows frank aspiration in roughly 20–25% of patients and substantial rates of laryngeal penetration and pharyngeal residue: for example, the five-year survivor cohort reported 24% tracheal aspiration and 36% laryngeal penetration on FEES with pharyngeal residue in ~72% of patients, while Bhattacharya’s Video-fluoroscopy (VFS)-based study found abnormal penetration aspiration in ≈20% and oral-phase abnormalities and residue as the most common deficits. These objective impairments often coexist with the patient’s adaptation: many survivors report acceptable global quality of life scores despite measurable aspiration or restricted diets, highlighting that objective dysfunction and patient-perceived quality can diverge and that individualized rehabilitation remains essential [3,49,50,51]. Patient-reported quality of life data, though limited, demonstrate this paradox: in a single-institution case series of TGTL survivors, mean University of Washington QoL score was 70/100—suggesting acceptable overall quality of life—yet the function sub score was markedly reduced at 36.4/100, while satisfaction with the surgical decision remained high at 4.4/5, reflecting complex trade-offs between oncologic control and functional impairment [52,53].

Reconstructive strategy, specifically the “mount” or bulk and shape of the neo-tongue, is a major determinant of functional outcome [54,55,56] (Figure 7b). Multiple studies and reviews emphasize that a bulky, projecting neo-tongue that provides palatoglossal contact and a propulsive surface yields better speech articulation and oral-phase propulsion, while thin or rapidly atrophying flaps (or inadequate initial volume) correlate with poor bolus control, more residue, and greater PEG dependence. Authors therefore recommend reconstruction with reliably voluminous flaps (rectus abdominis, ALT, latissimus when appropriate), planned overcorrection (20–30%) to allow for postop shrinkage, and adjuncts such as laryngeal suspension or delayed fat grafting/palatal augmentation when needed to improve palatoglossal contact and reduce aspiration [50,54,55,56,57,58]. Novel reconstruction techniques continue to emerge, including the ghost-shaped anterolateral thigh flap specifically designed for total tongue reconstruction, which aims to optimize neo-tongue volume and contour [59]. Taken together, flap choice, initial bulk, and planned overcorrection are modifiable factors that materially affect the patient’s ability to eat and speak, with evolving robotic-assisted flap inset potentially offering enhanced precision for reconstructing complex three-dimensional defects in the posterior oral cavity and oropharynx.

Studies use a combination of objective instrumental tests and validated scales. VFS with measures such as oral transit time (OTT), oral residue (OR), pharyngeal transit time (PTT) and the Rosenbek penetration–aspiration (P/A) scale; fiber-optic endoscopic evaluation of swallowing (FEES) often reporting penetration/aspiration and residue; challenge tests such as the 100 mL water swallow; and functional scales including the Functional Oral Intake Scale (FOIS) for diet level, the London Speech Evaluation Scale and Speech Handicap Index (SHI) for speech, and patient-reported QOL instruments (EORTC QLQ-C30/HN35, PSS-HN) [60,61,62,63,64,65,66,67]. Using both objective measures and patient-reported outcome measures (PROMs) allows the clinician to quantify physiologic impairments while also capturing the patient’s perceived function; both are needed to guide reconstruction and rehabilitation. A comprehensive review of swallowing indices is beyond the scope of this manuscript. The limitation of this meta-analysis is that PROM outcomes or quality-of-life measures were not included; incorporating these would provide a more comprehensive understanding of functional recovery and should be considered in future research.

Importantly, the potential for intensive rehabilitation to improve outcomes has been demonstrated even in this challenging patient population. A case report of transcranial direct current stimulation (tDCS) combined with speech therapy in a total glossectomy patient showed measurable improvements in speech articulation (percentage of consonants correct increased by 7% in naming tasks; spontaneous speech improved from 96% to 99.2%), alongside gains in quality of life measures (MD Anderson Dysphagia Inventory increased from 48 to 63, Speech Handicap Index improved), suggesting that adjunctive neurostimulation techniques may augment traditional rehabilitation strategies [68].

TG is among the most extensive procedures in head and neck oncology, and its execution demands not only surgical expertise but also robust rehabilitative infrastructure. In centers where reconstructive and speech/swallowing rehabilitation services are unavailable, offering this surgery is inappropriate, as long-term outcomes hinge on multidisciplinary support. Beyond the anticipated deficits in deglutition and communication, clinicians must recognize additional challenges such as orthopnea, which may result from the reconstructed bulk falling posteriorly into the pharynx, further compromising airway patency. This burden is exacerbated by the presence of a tracheostomy tube, complicating patient positioning and tolerance during adjuvant radiotherapy, particularly in the supine posture required for several minutes. Such complexities must be clearly communicated during preoperative counseling, underscoring that the objective of treatment extends beyond oncological clearance to encompass functional recovery and quality of life. Comprehensive rehabilitation addressing airway, nutrition, and communication is therefore indispensable to achieving meaningful outcomes in this patient population.

### 4.4. Voice Banking to Improve the QOL

Voice banking (also sometimes called “voice conservation”) refers to the process of pre-treatment (or pre-laryngectomy) recording of one’s natural voice to build a personalized synthetic voice (PSV) or text-to-speech (TTS) model that approximates the individual’s voice. This approach has been trialed in patients undergoing laryngectomy, where pre-laryngectomy recordings allow for preservation of a familiar-sounding synthetic voice. Its potential application to patients undergoing total glossectomy, however, remains largely unexplored. Given that functional speech outcomes are often poor and tracheostomy dependence is not uncommon in this cohort, particularly among frail or elderly patients. In such individuals, preservation of vocal identity through preoperative voice recordings and AI-driven text-to-speech cloning could provide a valuable means of maintaining personal communication when natural speech is severely compromised or absent [69,70,71].

An unpublished pilot study from the primary author’s institution explored the feasibility of voice banking and AI-driven text-to-speech cloning in patients undergoing major oral cavity resections with free flap reconstruction. Thirty patients were recruited between January and March 2025, including maxillectomy (*n* = 8), composite resections (*n* = 14), and hemiglossectomy (*n* = 8). Pre-treatment voice recordings were collected, and patient-specific speech models were generated using Resemble.ai tools. Voice similarity (A score) was assessed at baseline, three months, and six months. Across subsites, mean similarity scores improved over time, with glossectomy patients demonstrating slightly lower but still favorable results. Importantly, all patients and their relatives correctly recognized the AI-generated voice as their own, and acceptance of the cloned voice was universal. These findings suggest that voice banking is technically feasible, well-accepted by patients, and capable of preserving vocal identity during periods of impaired speech. The authors conclude that larger multicenter studies with extended follow-up are needed to establish the role of this technology in head and neck oncology.

### 4.5. Decision-Making in Advanced Tongue Cancer

**Figure 8:**
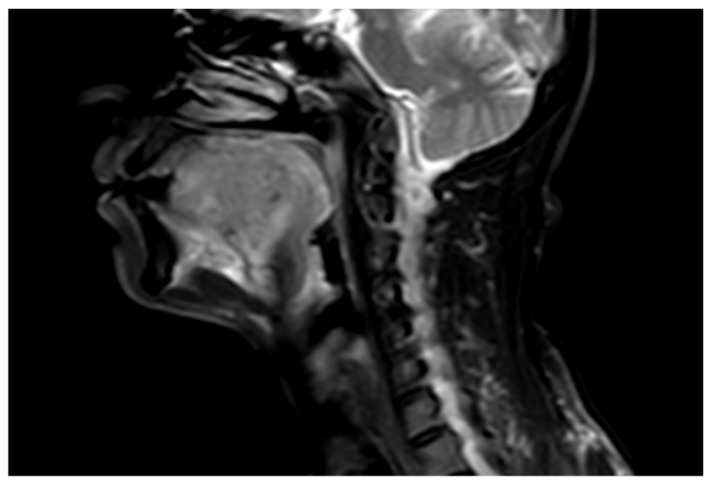
Sagittal CEMRI reveals a T2 hyperintense enhancing lesion with epiglottic involvement, a key diagnostic feature that defines the tumor’s extent. The absence of a clear surgical margin is prognostically critical, as it dictates the necessity for a more extensive resection, such as a laryngectomy, to achieve oncologic control.

The decision to proceed with a TG must carefully integrate the tumor, patient, and facility-specific factors, recognizing that oncologic control cannot be meaningfully pursued in isolation from postoperative function and rehabilitation potential. From the oncologic standpoint, several tumor characteristics argue strongly against total glossectomy. First, the adequacy of the mucosal margin is paramount: when the distance between the distal edge of the ulcer and the epiglottis is less than 1 cm after accounting for palpable induration, achieving a true mucosal clearance of 5 to 7 mm may not be feasible [33]. Such assessment can only be made by the operating surgeon through careful palpation, often necessitating examination under anesthesia, since severe ankyloglossia and pain frequently limit reliable outpatient evaluation. Cross-sectional imaging, particularly contrast-enhanced MRI of the neck, should complement the clinical assessment to detect subtle submucosal extension or epiglottic involvement that may escape palpation, including the well-recognized phenomenon of tumor islands in tongue carcinomas (Figure 8). Importantly, if the epiglottis itself is involved, the patient becomes a candidate for glosso-laryngectomy—a highly morbid procedure that is rarely performed due to its significant morbidity, long-term tube dependence, and poor functional outcomes.

Second, involvement or erosion of the hyoid bone represents a critical determinant of resectability (candidate for glosso-laryngegctomy). Tumor arising from the floor of mouth or adjacent neurovascular structures may extend submucosally to the hyoid, and careful bi-digital palpation—placing one finger in the vallecula and applying counter-pressure on the hyoid—remains a simple yet essential technique to evaluate such a disease. A bulky lesion that prevents the examiner’s fingers from approximating is strongly suggestive of hyoid-level involvement. Some centers advocate shaving the hyoid or even performing partial resection to achieve negative margins, but the long-term oncologic benefit of this strategy remains uncertain and warrants further study.

Third, locally advanced ulcers that extend laterally to erode the mandible require composite resections, often necessitating segmental mandibulectomy in addition to total glossectomy. While such disease is not necessarily inoperable, the ablative burden is considerable, creating two distinct anatomical subunits to reconstruct rather than one. These cases pose formidable challenges to the reconstructive surgeon, and functional outcomes are generally poor, with long-term dependence on enteral nutrition and airway support being common sequelae.

**Figure 9:**
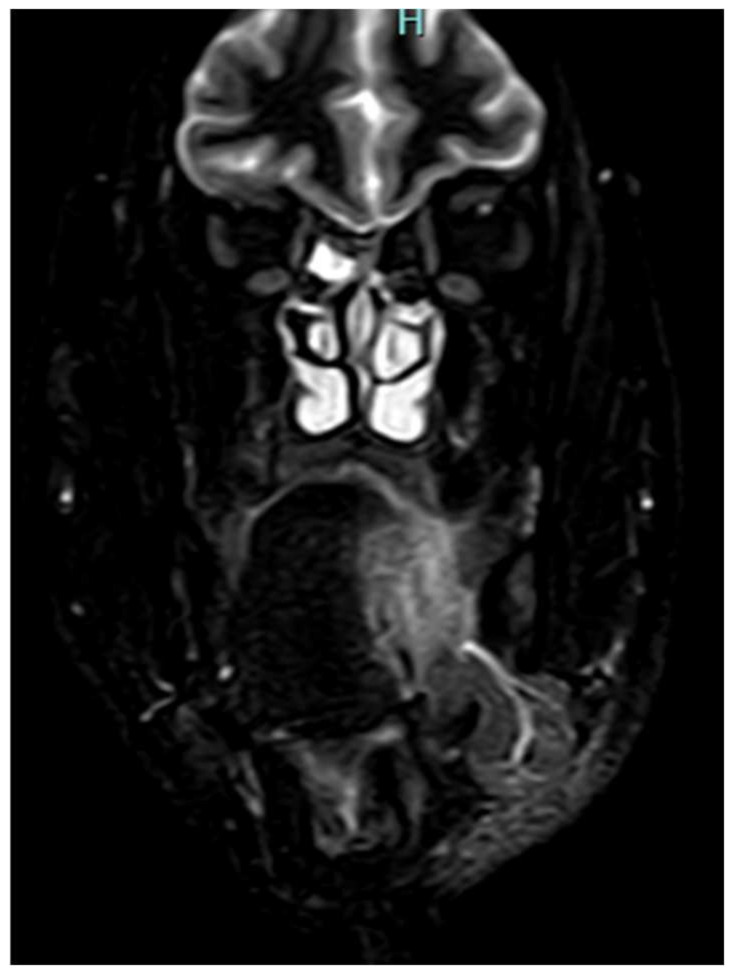
Coronal magnetic resonance imaging (MRI), demonstrating a key diagnostic finding of lateral extension of the primary lesion into the tonsil and tonsillo-lingual sulcus, resulting in a confluent mass involving both the tumor and an ipsilateral N3b nodal metastasis (node–primary complex).

Finally, lateral extension of the primary lesion into the tonsil or tonsillo-lingual sulcus may create a confluent mass incorporating both the tumor and an ipsilateral N3b nodal metastasis, forming a so-called node–primary complex. This pattern of spread represents a biologically aggressive disease state and portends one of the worst prognoses. In such scenarios, neoadjuvant chemotherapy may be considered as an attempt to downstage disease before contemplating radical surgery, although the likelihood of durable disease control remains limited (Figure 9).

### 4.6. Fate of TG in the Era of Neoadjuvant Immunotherapy

The advent of neoadjuvant immunotherapy is reshaping the management of locally advanced tongue cancer and compels a re-examination of the role of total glossectomy [72]. Patients with resectable stage III–IVA disease, who historically underwent this most disabling of operations, are now eligible for perioperative pembrolizumab if the combined proportion score (CPS) is ≥1, with the greatest benefit observed in those with CPS ≥10. Response rates to checkpoint blockade include a subset achieving major or even complete pathologic responses, raising the question of whether ablative surgery is mandatory in all cases [73,74]. At present, surgery remains the standard, yet the scenario of a radiologic or clinical complete response challenges conventional sequencing and revives debate regarding the appropriateness of radiation or chemoradiation alone. The future of TG will depend on biomarker-driven trials that can define which patients may forgo surgery without compromising oncologic outcomes. Until such evidence emerges, the procedure must be considered within a multidisciplinary framework, with careful attention to the balance between disease eradication and preservation of function [75,76]. In the event of a complete clinical and radiological response (CCR) following treatment with anti-PD1 agents, the decision to proceed with radical chemoradiotherapy (CRT) or to undertake a morbid surgery—one that will almost certainly result in significant functional debilitation—still requires stronger evidence and careful evaluation. When the CPS is favorable, the rationale for aggressive surgical intervention becomes even more debatable. It is therefore essential that patients receive thorough counseling: they must be informed that, despite achieving CCR, surgery may not achieve the expectation of a pathological complete response. The patient’s willingness to undergo such a procedure, fully understanding both the potential benefits and the likelihood of long-term functional compromise, must be clearly established [77,78,79,80].

## 5. Conclusions

In this proportional meta-analysis of upfront TG followed by adjuvant therapy, pooled survival declined from a 1-year overall survival of 79% to 55% at 3 years and 47% at 5 years, with the more recent series (post-2000) showing improved 5-year survival (~63%). Outcomes are heavily influenced by indication and stage: patients undergoing primary TG have markedly better oncologic outcomes than those with salvage TG after failed organ-preservation therapy, and advanced T-stage, nodal burden, and positive/close margins, which remain dominant adverse prognostic factors. Functional morbidity is frequent and clinically meaningful—although most survivors regain at least partial oral intake, a substantial minority remain gastrostomy-dependent or aspirate (objective aspiration ~20–25%), and prolonged tracheostomy dependence is more likely after extensive or salvage resections. Reconstructive strategy and multidisciplinary rehabilitation are, therefore, critical and modifiable determinants of long-term function; achieving and maintaining sufficient neo-tongue bulk, planned flap overcorrection, laryngeal suspension, and early, specialized rehabilitation consistently improve swallowing and speech outcomes, and adjuncts such as voice banking and AI voice cloning show promise for mitigating communication loss. The advent of neoadjuvant immunotherapy, which produces pathologic responses and improves event-free survival in oral-cavity SCC, compels a reassessment of TG’s role, but de-escalation of surgery should be restricted to biomarker-defined, trial-eligible patients until prospective evidence supports omission [81,82]. Going forward, multicenter, prospective studies and standardized reporting of both oncologic and functional endpoints are urgently needed to define which patients may safely avoid or undergo less extensive resections while preserving survival and quality of life.

## Figures and Tables

**Table 1:** Summary of Studies Included in TG Meta-Analysis. OS = Overall Survival; DFS = Disease-Free Survival; N = Sample size; NOS = Newcastle–Ottawa Scale; NR = Not Reported; TGLAP = TG with Laryngeal and Pharyngeal Preservation; PEG = Percutaneous Endoscopic Gastrostomy; - = Data not available.

Study	Year	Country	NOS	No. of Patients (N)	1-Yr OS (%)	1-Yr DFS (%)	3-Yr OS (%)	3-Yr DFS (%)	5-Yr OS (%)	5-Yr DFS (%)	Oral Feeding Details	Tracheostomy Details	Speech Details
Gehanno et al. [18]	1992	USA	7/9	32	75	-	47	-	12	-	NR	NR	NR
Magrin et al. [19]	1996	Brazil	7/9	75	-	-	-	-	29	-	NR	NR	NR
Ruhl et al. [20]	1997	USA	7/9	20	-	-	55	-	45	-	NR	NR	NR
Bova et al. [21]	2004	Australia	7/9	14	93	93	-	-	29	29	90% achieved independent oral feeding before discharge	6/7 TGLAP patients successfully decannulated (86%)	57% of TGLAP patients achieved satisfactory vocal rehabilitation
Sinclair et al. [22]	2011	USA	8/9	20	-	40	-	-	-	-	0% complete independence; 33% partial; 70% total PEG dependence	50% remained tracheostomy dependent	30% achieved functional speech
Vega et al. [23]	2011	Spain	6/9	12	92	-	67	-	67	-	85% resumed oral feeding (64% good, 21% acceptable diet)	90% of non-laryngectomy patients decannulated	87% of non-laryngectomy patients achieved good/acceptable speech
Navach et al. [24]	2013	Italy	8/9	24	-	-	-	-	79	61	70% eventually returned to oral feeding (16 pureed, 10 resumed after PEG)	Not specified	100% recovered intelligible speech
Reiter et al. [25]	2017	Germany	8/9	7	86	-	57	-	-	-	79% (11/14) resumed oral feeding without gastrostomy	86% (12/14) permanently decannulated	86% (12/14) achieved good/acceptable speech
Han et al. [26]	2019	USA	8/9	20	60	-	30	-	30	-	NR	NR	NR
Huang et al. [27]	2022	Taiwan	7/9	37	84	64	73	57	73	57	NR	NR	NR

## Data Availability

No new data were created or analyzed in this study. Data sharing is not applicable to this article.

## Supplementary Materials

The following supporting information can be downloaded at https://www.mdpi.com/article/10.3390/diagnostics15222847/s1. Table S1: Structure of the Electronic Search Strategy; PRISMA 2020 Checklist. Reference [83] is cited in the Supplementary Materials.
