# Supplementary material for: Prognostic Value of Primary Total Glossectomy in Tongue Cancer: A Systematic Review and Meta-Analysis of Survival Outcomes
Source: Diagnostics (Basel). 2025 Nov 10;15(22):2847. doi: 10.3390/diagnostics15222847 (PMC12651312; doi:10.3390/diagnostics15222847)
Supplement: Supplementary file 1 [file diagnostics-15-02847-s001.zip › diagnostics-3954626-PRISMA 2020 Checklist.pdf]

PRISMA 2020 Checklist

Section and Topic	Item #	Checklist item	Location where item is reported
**TITLE**			
Title	1	Identify the report as a systematic review.	Title (includes "meta-analysis")
**ABSTRACT**			
Abstract	2	See the PRISMA 2020 for Abstracts checklist.	Abstract section with Background, Methods, Results, Conclusions
**INTRODUCTION**			
Rationale	3	Describe the rationale for the review in the context of existing knowledge.	Introduction, paragraphs 1-3
Objectives	4	Provide an explicit statement of the objective(s) or question(s) the review addresses.	Introduction, final paragraph
**METHODS**			
Eligibility criteria	5	Specify the inclusion and exclusion criteria for the review and how studies were grouped for the syntheses.	"Study Selection and Eligibility Criteria" section; "Studies were excluded if..." paragraph
Information sources	6	Specify all databases, registers, websites, organisations, reference lists and other sources searched or consulted to identify studies. Specify the date when each source was last searched or consulted.	"Literature Search Strategy" section: "PubMed, Scopus and Google Scholar...to June 26, 2025"
Search strategy	7	Present the full search strategies for all databases, registers and websites, including any filters and limits used.	"Literature Search Strategy" section: search terms listed (glossectomy, total glossectomy, survival, etc.)
Selection process	8	Specify the methods used to decide whether a study met the inclusion criteria of the review, including how many reviewers screened each record and each report retrieved, whether they worked independently, and if applicable, details of automation tools used in the process.	"Study Selection and Eligibility Criteria" section: "Two independent reviewers (PD and SMP)...disagreements resolved by third reviewer (KNR)"
Data collection process	9	Specify the methods used to collect data from reports, including how many reviewers collected data from each report, whether they worked independently, any processes for obtaining or confirming data from study investigators, and if applicable, details of automation tools used in the process.	"Data Extraction Process" section: "Two independent reviewers (PD and

Section and Topic	Item #	Checklist item	Location where item is reported
			SMP)...third reviewer (KNR)"
Data items	10a	List and define all outcomes for which data were sought. Specify whether all results that were compatible with each outcome domain in each study were sought (e.g. for all measures, time points, analyses), and if not, the methods used to decide which results to collect.	"Data Extraction Process" section: OS and DFS at 1, 3, and 5 years
	10b	List and define all other variables for which data were sought (e.g. participant and intervention characteristics, funding sources). Describe any assumptions made about any missing or unclear information.	"Data Extraction Process" section: patient demographics, TNM staging, surgical approach, reconstruction methods
Study risk of bias assessment	11	Specify the methods used to assess risk of bias in the included studies, including details of the tool(s) used, how many reviewers assessed each study and whether they worked independently, and if applicable, details of automation tools used in the process.	"Methodological Quality" section: Newcastle-Ottawa Scale (NOS), two independent reviewers
Effect measures	12	Specify for each outcome the effect measure(s) (e.g. risk ratio, mean difference) used in the synthesis or presentation of results.	"Primary Analysis" section: pooled survival proportions with 95% confidence intervals
Synthesis methods	13a	Describe the processes used to decide which studies were eligible for each synthesis (e.g. tabulating the study intervention characteristics and comparing against the planned groups for each synthesis (item #5)).	"Study Selection and Eligibility Criteria" section
	13b	Describe any methods required to prepare the data for presentation or synthesis, such as handling of missing summary statistics, or data conversions.	"Data Extraction Process" section: Kaplan-Meier curve digitization when needed
	13c	Describe any methods used to tabulate or visually display results of individual studies and syntheses.	"Effect Size Visualization" section: Forest plots, L'Abbé plots
	13d	Describe any methods used to synthesize results and provide a rationale for the choice(s). If meta-analysis was performed, describe the model(s), method(s) to identify the presence and extent of statistical heterogeneity, and software package(s) used.	"Primary Analysis" and "Heterogeneity Assessment" sections: random-effects meta-analysis, DerSimonian-Laird, Cochran Q-test, I^2 statistic

Section and Topic	Item #	Checklist item	Location where item is reported
	13e	Describe any methods used to explore possible causes of heterogeneity among study results (e.g. subgroup analysis, meta-regression).	"Sensitivity and Subgroup Analyses" section: subgroup by publication period and sample size
	13f	Describe any sensitivity analyses conducted to assess robustness of the synthesized results.	"Sensitivity and Subgroup Analyses" section: leave-one-out analysis
Reporting bias assessment	14	Describe any methods used to assess risk of bias due to missing results in a synthesis (arising from reporting biases).	"Publication Bias Evaluation" section: funnel plots with note about limited power (n=10)
Certainty assessment	15	Describe any methods used to assess certainty (or confidence) in the body of evidence for an outcome.	"Certainty of Evidence Assessment" section in Methods describes that formal GRADE was not performed; quality evaluated based on NOS scores, consistency, precision, and heterogeneity
**RESULTS**			
Study selection	16a	Describe the results of the search and selection process, from the number of records identified in the search to the number of studies included in the review, ideally using a flow diagram.	"Results section - 'Literature Retrieval and Data Extraction' states: 'Seven studies were excluded during full-text review due to insufficient survival data, inability to separate TG outcomes from mixed cohorts, or failure to meet methodological quality standards.' Detailed records of excluded studies were not maintained."
	16b	Cite studies that might appear to meet the inclusion criteria, but which were excluded, and explain why they were excluded.	
Study characteristics	17	Cite each included study and present its characteristics.	Results section - "Study Characteristics

Section and Topic	Item #	Checklist item	Location where item is reported
			and Quality Assessment" subsection. Table 1 presents complete characteristics of all 10 included studies (Gehanno 1992, Magrin 1996, Ruhl 1997, Bova 2004, Sinclair 2011, Vega 2011, Navach 2013, Reiter 2017, Han 2019, Huang 2022). Studies cited as References 18-27.
Risk of bias in studies	18	Present assessments of risk of bias for each included study.	Results section - "Study Characteristics and Quality Assessment" subsection states: "Eight studies (80%) achieved high quality ratings (≥7 stars), while two studies (20%) received moderate quality ratings (4-6 stars)". Table 1 NOS column shows individual scores for each study (ranging 6/9 to 8/9).
Results of individual studies	19	For all outcomes, present, for each study: (a) summary statistics for each group (where appropriate) and (b) an effect estimate and its precision (e.g. confidence/credible interval), ideally using structured tables or plots.	Table 1 presents individual study survival rates at 1, 3, and 5 years with sample sizes. Figure 3 (A, B, C) shows forest plots with individual study estimates and 95% confidence intervals for 1-year, 3-year, and 5-year OS. Individual study ranges reported in text (e.g., "60% to 93%" for 1-year).

Section and Topic	Item #	Checklist item	Location where item is reported
Results of syntheses	20a	For each synthesis, briefly summarise the characteristics and risk of bias among contributing studies.	Results section. "One-Year Overall Survival" subsection: "6 studies encompassing 122 patients". "Three-Year Overall Survival" subsection: "6 studies including 128 patients". "Five-Year Overall Survival" subsection: "7 studies comprising 220 patients". Quality assessment summary provided earlier in Results.
	20b	Present results of all statistical syntheses conducted. If meta-analysis was done, present for each the summary estimate and its precision (e.g. confidence/credible interval) and measures of statistical heterogeneity. If comparing groups, describe the direction of the effect.	Results section presents: 1-year OS: 81% (95% CI: 71-90%), I^2=40.2%, τ^2=0.0095; 3-year OS: 55% (95% CI: 41-68%), I^2=58.4%, τ^2=0.0167; 5-year OS: 47% (95% CI: 27-67%), I^2=89.0%, τ^2=0.0668; Post-2000 analysis 5-year OS: 64% (95% CI: 39-83%). All presented in Figure 3 (A, B, C).
	20c	Present results of all investigations of possible causes of heterogeneity among study results.	Results section - "Heterogeneity Assessment and L'Abbé Plot Analysis" subsection with Figure 6 (A, B, C). "Post-2000 Sensitivity Analysis" subsection with Figure 5 shows improved 5-year OS (64% vs 47%) when excluding pre-2000 studies. Text discusses progressive

Section and Topic	Item #	Checklist item	Location where item is reported
			increase in heterogeneity over time.
	20d	Present results of all sensitivity analyses conducted to assess the robustness of the synthesized results.	Results section - "Sensitivity Analysis" subsection with Figure 4 presents leave-one-out analysis showing pooled estimates ranging 40-55% with maximum 8 percentage point difference. "Post-2000 Sensitivity Analysis" subsection with Figure 5 presents separate analysis by publication era showing improved outcomes in recent studies.
Reporting biases	21	Present assessments of risk of bias due to missing results (arising from reporting biases) for each synthesis assessed.	Results section - "Publication Bias Assessment" subsection with Figure 2 (A, B, C) showing funnel plots for 1-year, 3-year, and 5-year survival. States: "studies reasonably distributed around pooled estimates without systematic asymmetry" and "no evidence of publication bias despite substantial heterogeneity". Acknowledges limitation: "limited by the small number of included studies (n=10)".
Certainty of	22	Present assessments of certainty (or confidence) in the body of evidence for each outcome assessed.	"Results section -

Section and Topic	Item #	Checklist item	Location where item is reported
evidence			'Certainty of Evidence' subsection. States: 'The overall certainty of evidence was judged to be low to moderate for survival outcomes' due to retrospective design, substantial heterogeneity (I^2=89%), and small sample sizes. Consistency of effect and robust sensitivity analyses partially mitigated concerns."
**DISCUSSION**			
Discussion	23a	Provide a general interpretation of the results in the context of other evidence.	Discussion section - "Survival outcomes" subsection compares pooled results (81% 1-year, 55% 3-year, 47% 5-year OS) with individual study findings. Discusses individual studies (Bova 93% 1-year vs Gehanno 12% 5-year). "Prognostic Significance of Treatment Indication and Disease Stage" subsection contextualizes findings (primary vs salvage, T-stage impact). References recent cohort study: "3-year overall survival of 49.2%".
	23b	Discuss any limitations of the evidence included in the review.	"Discussion section - 'Disease-Free Survival' subsection notes 'limited reporting of DFS outcomes (60% did

Section and Topic	Item #	Checklist item	Location where item is reported
			not report DFS)'. 'Functional Outcomes' subsection states 'most studies did not separate outcomes for primary versus salvage surgery cases' and 'heterogeneity in outcome definitions precluded meta-analysis of functional endpoints'."
	23c	Discuss any limitations of the review processes used.	"Methods section - 'Publication Bias Evaluation' acknowledges 'limited by small number of studies (n=10)' and 'statistical tests have limited power with <15 studies'. Protocol was not registered. English-language restriction applied. Individual patient data not available."
	23d	Discuss implications of the results for practice, policy, and future research.	Discussion section - "Decision making in advanced tongue cancer" subsection provides practice implications (tumor factors, patient selection, surgical technique considerations). States: "TG survivors demonstrate wide spectrum...substantial minority experience persistent aspiration risk" and "requires careful multidisciplinary deliberation including

PRISMA 2020 Checklist

Section and Topic	Item #	Checklist item	Location where item is reported
			tumor board, reconstructive and rehabilitation teams". "Fate of TG in the era of neoadjuvant immunotherapy" subsection discusses future research. Conclusion states: "multicenter, prospective studies and standardized reporting of both oncologic and functional endpoints are urgently needed" and advocates for "biomarker-driven trials".
**OTHER INFORMATION**			
Registration and protocol	24a	Provide registration information for the review, including register name and registration number, or state that the review was not registered.	"Methods section - 'Protocol and Registration' subsection explicitly states: 'This systematic review and meta-analysis was not prospectively registered in a public registry such as PROSPERO or Open Science Framework.'"
	24b	Indicate where the review protocol can be accessed, or state that a protocol was not prepared.	"Methods section - 'Protocol and Registration' subsection states: 'The review was conducted according to a predefined protocol developed collaboratively by the review team, though this protocol was not formally published or made publicly available prior to

PRISMA 2020 Checklist

Section and Topic	Item #	Checklist item	Location where item is reported
			study commencement.'"
	24c	Describe and explain any amendments to information provided at registration or in the protocol.	"Not applicable - review was not registered"
Support	25	Describe sources of financial or non-financial support for the review, and the role of the funders or sponsors in the review.	"Title page states: 'No Funding'. This research received no specific grant from any funding agency."
Competing interests	26	Declare any competing interests of review authors.	"Title page states: 'No conflict of Interest'. The authors declare no competing interests."
Availability of data, code and other materials	27	Report which of the following are publicly available and where they can be found: template data collection forms; data extracted from included studies; data used for all analyses; analytic code; any other materials used in the review.	"Data Availability Statement section (before References) states: 'All data extracted from included studies are presented within this manuscript (Table 1 and Figures 2-6). Individual patient-level data were not available. Data extraction forms, NOS assessment records, and R code (version 4.5.1) available from corresponding author upon reasonable request.'"

From: Page MJ, McKenzie JE, Bossuyt PM, Boutron I, Hoffmann TC, Mulrow CD, et al. The PRISMA 2020 statement: an updated guideline for reporting systematic reviews. BMJ 2021;372:n71. doi: 10.1136/bmj.n71. This work is licensed under CC BY 4.0. To view a copy of this license, visit https://creativecommons.org/licenses/by/4.0/
