# Supplementary material for: Prognostic Value of Primary Total Glossectomy in Tongue Cancer: A Systematic Review and Meta-Analysis of Survival Outcomes
Source: Diagnostics (Basel). 2025 Nov 10;15(22):2847. doi: 10.3390/diagnostics15222847 (PMC12651312; doi:10.3390/diagnostics15222847)
Supplement: Supplementary file 1 [file diagnostics-15-02847-s001.zip › diagnostics-3954626-Table S1.pdf]

Table – Supplementary material

Table: Structure of the Electronic Search Strategy

Search Component	Key Concepts & Search Terms
**Population / Intervention**	"glossectomy" OR "total glossectomy" OR "complete glossectomy" OR "tongue resection" OR "oral cancer surgery" OR "tongue cancer"
**Outcomes**	"survival" OR "mortality" OR "prognosis" OR "follow-up" OR "overall survival" OR "disease-free survival"
**Final Search Query**	**(Population/Intervention Terms) AND (Outcome Terms)**
